# Type 4 capitellum fractures: Diagnosis and treatment strategies

**DOI:** 10.4103/0019-5413.53460

**Published:** 2009

**Authors:** SS Suresh

**Affiliations:** Department of Orthopaedics, Ibri Regional Referral Hospital, PO Box 46, Ibri 516, Sultanate of Oman

**Keywords:** Capitellum fracture, type 4 capitellum fracture, bone fixation, bone screw

## Abstract

**Background:**

Fractures of the capitellum are rare injuries of the elbow usually seen in the adolescents. This fracture is often missed in the emergency room if a proper radiograph is not available. Recent reports have described many modalities of treatment favoring headless screw for fixation. The facility for headless screw fixation, however, is not available in most centers. This paper presents the diagnosis and management of type 4 capituller fractures (Mckee) with gadgets available in a district hospital.

**Materials and Methods:**

Between 2004 and 2007 three patients with right sided type IV capetullar fracture were treated in a district hospital. There were two boys aged 15 and 17 and one 33 years old lady. In one case, the fracture was missed in the emergency room. A double arc sign in the lateral views of the X-rays of the elbow was seen in all the cases. In each case a preoperative CT scan was done and a diagnosis of Mckee type IV fracture of the capitellum was made. Under tourniquet, using extended lateral approach, open reduction and internal fixation was done using 4mm partially threaded AO cancellous screws (n=2) and 2.7 mm AO screws (n=1), under vision from posterior to anterior direction from the posterior aspect of lateral condyle of humerus avoiding articular penetration.

**Results:**

All the fractures united uneventfully. At the end of one year follow-up, two cases had excellent elbow function; implants were removed and there were no signs of AVN or arthritis. The third case had good elbow ROM at 11 months without AVN.

**Conclusion:**

Double arc sign on lateral X-rays of the elbow along with pre-operative CT scan evaluation is important to avoid a missed diagnosis and analysis of type IV capitellur fracture. Fixation with non-cannulated ordinary AO screws using extended Kocher's lateral approach has given good results.

## INTRODUCTION

Capitellum fractures are rare elbow injuries accounting for less than one per cent fractures around the elbow.^1^–^3^ A capitellum fracture is, usually, a fracture of adolescents with most cases occurring in children above 12 years of age.^4^,^5^

Treatment modalities vary from conservative, in the form of closed reduction and immobilization,^6^,^7^ fragment excision^2^,^7^,^8^ to open reduction and internal fixation with K wires^1^ and 4 mm partially threaded cancellous screws^9^ or Herbert screws.^10^,^11^

Open reduction and stable internal fixation helps in early mobilization, preventing stiffness of the elbow, and subsequent degenerative arthritis, as the articular congruity is maintained by anatomical reduction. Extension of the capitellar fracture well medially into the trochlea is reported^12^,^13^ and currently classified as the fourth type.^13^ This is also described as the coronal shear fracture. The fragment is intraarticular, usually, without any soft tissue attachments. If not properly treated it results in malunion interfering with flexion of the elbow. The fragment has to be anatomically reduced and properly stabilized to prevent articular incongruity and late onset arthritis. A proper classification helps in pre-operative planning and execution of the surgical stabilization.

As early as 1935, Mazel described capitellum fracture as a layer of bone with a portion of trochlea attached to it.^7^ Capitellar fractures are classified into three types. Type 1 (Hahn-Steinthal fracture) which consists of a large fragment of cancellous bone of the articular surface of capitellum and may include a portion of the trochlea, typically the lateral third;^1^,^8^,^13^ type 2 fracture (Kocher-Lorenz fracture) which is cartilaginous articular fracture of the capitellum and may include a small fragment of sub-chondral bone typically described as “uncapping” of the capitellum;^14^ type 3 (Broberg and Morrey), a comminuted capitellar fracture.^13^,^15^

If the fracture extends to more than the lateral half of the trochlea it is considered a separate entity.^13^ Another fracture, described only in children, the “sleeve fracture,” has also been reported.^5^ As per AO classification, these will be classified as B3.1 (capitellar fractures), B3.2(trochlear) and B3.3 (capitellar and trochlear fractures).^9^ Dubberley^9^ classified capitellar fractures into three types, type 1 involving primarily the capitellum with or without lateral trochlear ridge, type 2 with fracture of capitellum and trochlea as one piece, and type 3 fractures of, both capitellum and trochlea, where the capitellum and trochlea are separate fragments. They further sub-classified these fractures depending on absence (A) and presence (B), of posterior condylar comminution.

The purpose of this paper is to present the diagnosis and management of three cases of type 4 fractures^13^ with gadgets available in a district hospital. All patients had the “double arc” sign which is diagnostic of type 4 injury.

## MATERIALS AND METHODS

Three patients were treated in a district hospital, from 2004 to 2007, for Type IV capitellar fracture. Two of them were adolescent boys aged 15 and 17, and the third, a 33-year-old lady. The mechanism of injury in two of the cases was fall from height, when they landed on an out-stretched hand. All fractures were on the right side, the dominant hand, in the patients.

In one case, the fracture was missed in the emergency room. A double arc sign was the finding, in the lateral view, of the X-rays of the elbow. CT scans of all three patients showed the extent of articular fracture components. All patients were found to have Mc Kee Type IV fracture of the capitellum (Dubberley 3).^9^,^13^ Routine radiographs and CT scans of the elbows were done in all cases. All patients were operated under general anaesthesia under tourniquet control through an extended lateral Kocher's approach. The extensor origin was elevated in all cases subperiosteally including the origin of the extensor carpi radialis longus. The origin of the lateral collateral ligamentous complex from the lateral epicondyle was not disturbed. The exposure is extended distally between the anconeus and the extensor carpi ulnaris. Keeping the forearm pronated the extensor carpi ulnaris is elevated anteriorly. This allows the surgeon to reflect the soft tissues to keep the bone levers over the medial column. The extensive exposure aided in keeping a bone lever over the medial aspect of the distal humerus, thus helping in visualization of the entire articular surface of the distal humerus. The fracture was reduced by checking the anterior articular surface, and held reduced with smooth K wires. Definitive stabilization was done with 4 mm partially threaded cancellous screws (two cases) and 2.7 mm screw (one case), from posterior to anterior direction from the posterior aspect of the lateral condyle. Since the articular surface was visible, penetration of the articular surface by the screw threads was avoided. The joint was irrigated and fragments of bone were removed. A Plaster of Paris (POP) slab was given in all cases with elbow at 90 degrees of flexion and the forearm in neutral rotation. The patients were mobilized out of back slab after three weeks. Range of motion exercise was started under supervision of physiotherapist after six weeks. Clinical and radiological follow up was done at six weeks, three months, six months and one year. The elbows were tested for range of movements, and instability. Radiographic assessment was done as per Broberg and Morrey grading 9,12 and none of the three patients were found to have evidence of arthritis, AVN or heterotopic ossification at the end of follow up

### Case 1

**Figure 1:**
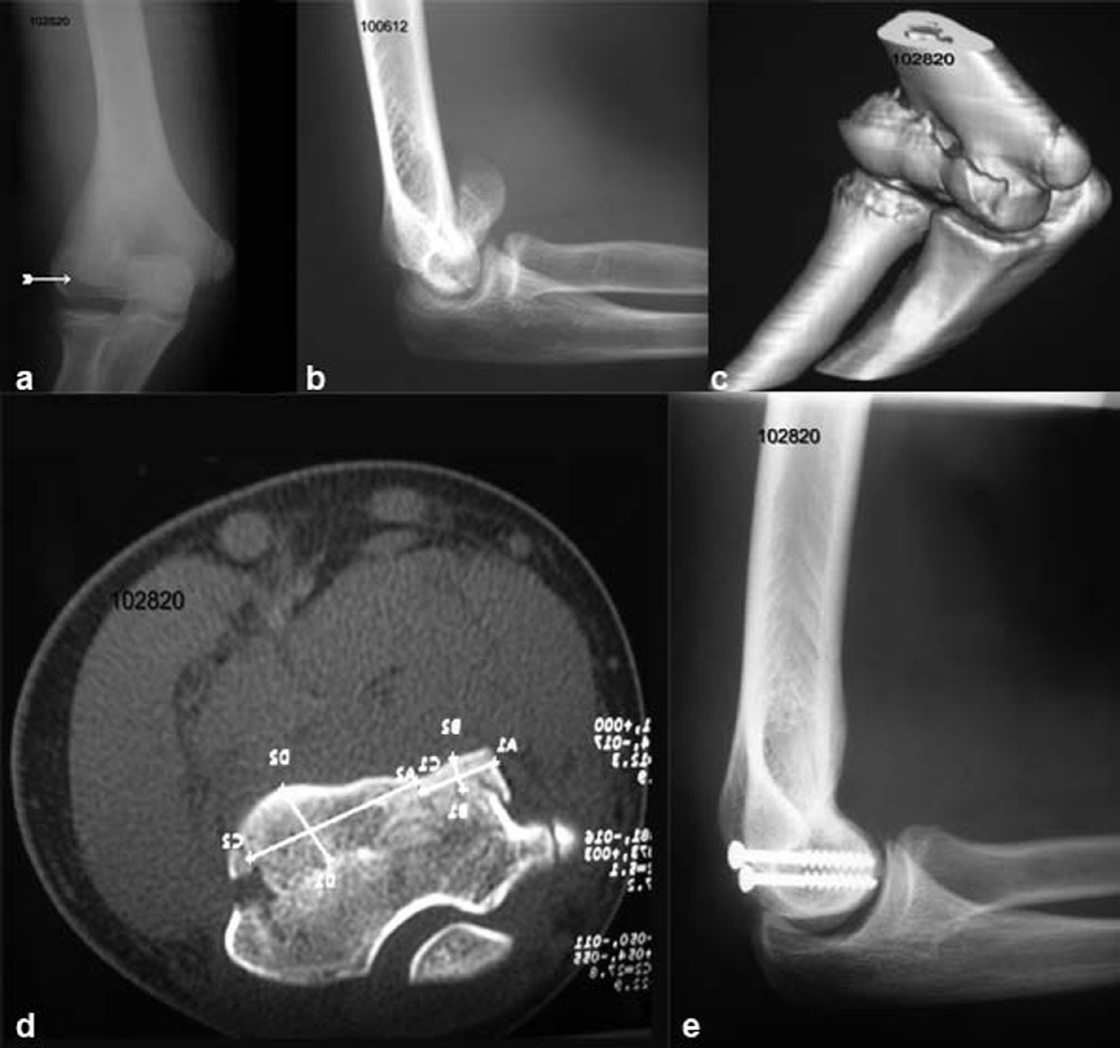
(a) Anteroposterior view of the elbow showing the fracture (white arrow) which is easily missed in emergency room. (b) True lateral view of elbow showing double arc sign. (c) 3D CT scan showing the involvement of entire trochlea. (d) Axial section of CT scan showing the measurements of the fragments. (e) Lateral X-ray of the elbow showing fixation with 4 mm cancellous screws

The first case (male, 15 years) sustained the injury after a fall from a motor cycle and the fracture was missed in the Accident and Emergency [Figure 1a]. Subsequently, he presented in the orthopaedic clinic, after 48 hours, with pain and swelling of the right elbow, and limitation of flexion. There was tenderness of the lateral aspect to the elbow. A true lateral view showed the “double-arc” sign [Figure 1b].

A CT scan of the elbow was confirmative and showed the fracture extending to the medial ridge of the trochlea [Figure 1c and d]. The patient underwent open reduction through an extended lateral Kocher's approach, and the fracture was fixed with two 4 mm partially threaded cancellous screws, from posterior to anterior. He regained a full range of movements and on follow up [Figure 1e] there were no radiological signs of arthritis or avascular necrosis (AVN).

The screws were removed after one year through stab incisions.

### Case 2

**Figure 2:**
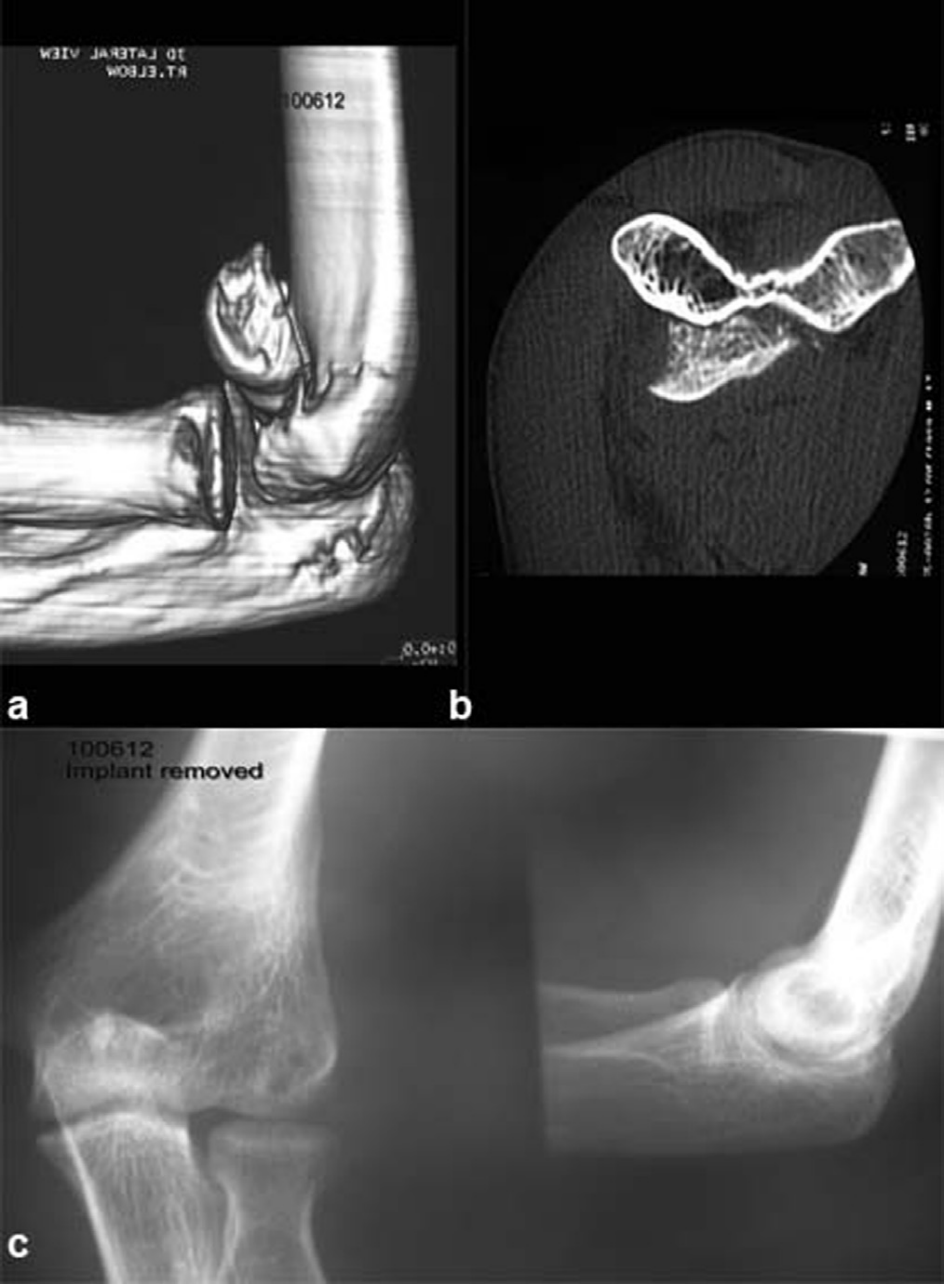
(a) 3D CT scan showing involvement of the trochlea. (b) CT scan axial section showing the involvement of the trochlea upto the trochlear groove. (c) X-rays after implant removal showing normal articular congruity

The second case, a male of 17 years, sustained the injury in a fall from height, after he fell on the out-stretched hand. X-rays were confirmative showing the “double arc” sign. Delineation of the fracture geometry was done with a CT scan, which showed comminution of the articular surface [Figure 2a and b].

He underwent open reduction through an extensile lateral kocher approach and fixation was done with 4 mm partially threaded cancellous screws (Synthes AO). He had an extension lag of 10 degrees, but no evidence of arthritis or AVN, at one year follow-up. The implants were removed after one year. The patient had 10 degrees of extension lag, with full flexion at one year follow-up [Figure 2c].

### Case 3

**Figure 3:**
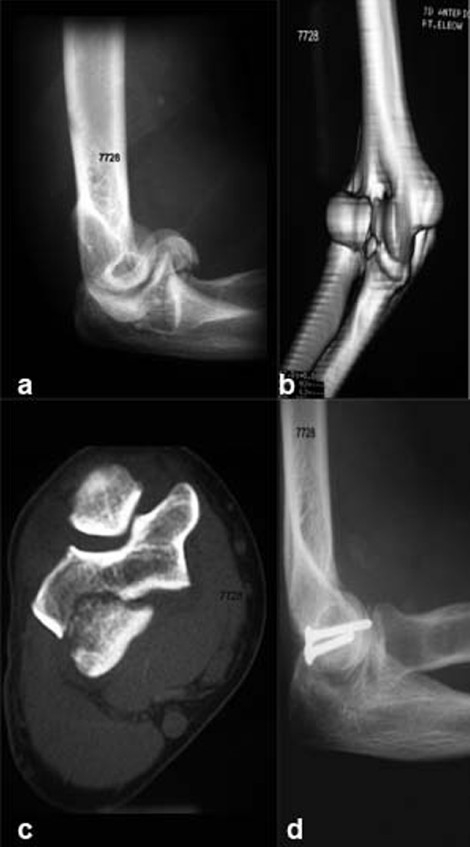
(a) X-ray showing double arc sign, fragments displaced distally. (b) 3D CT scan showing the involvement of the lateral trochlear ridge. (c) Axial section of CT scan showing the lateral trochlear ridge fracture. (d) Lateral X-ray of the elbow showing fixation with 2.7 mm screws

A 33-year-old lady had a fall and sustained fracture of the right elbow. The fracture was found displaced inferiorly, with double arc sign [Figure 3a–d]. She could flex her elbow to 100 degrees in the emergency room. She underwent open reduction and internal fixation with 2.7 mm AO screws, through a lateral approach. At 11 month follow-up she recovered her function with eight degrees of extension lag, terminal five degrees of flexion deficit and full fore-arm function [Table 1].

**Table 1:** Clinical details of the patients

Age/Sex	MOI	X-ray	CT scan	Followup (months)	Fixation	Function
15/M	Motor cycle accident	Double arc sign	27 × 14 mm capitellum fragment, 13 × 9.5 mm trochlea fragment, superior displacement of 12 mm	17	4 mm cancellous screws	Full range of movements/no arthritis / Implant removed
17/M	Fall on out stretched hand	Double arc sign	Fragment of 28.5 mm × 11.5 mm consisting of capitellum and lateral part of trochlea. Displacement 15 mm	12	4 mm cancellous screws	10° extension lag/full flexion/no arthritis/implant removed
33/F	Fall on out stretched hand	Double arc sign	Capitellar and lateral trochlear ridge fragment	11	2.7 mm AO screws	8° extension lag/ flexion terminal 5°/ no arthritis

## DISCUSSION

Capitellum fractures are rare injuries that occur in adolescents over the age of 12 years.^1^–^5^ Though, reportedly, more common in females, with a male to female ratio of 1:4,^14^,^16^ two of the three cases in this series were males. Mechanism of injury is usually a fall on the out-stretched hand, the radius imparting a shearing force on the capitellum.^1^,^5^ Maximum force transmission through the radial head to the capitellum occurs at zero to thirty degrees of elbow flexion.^17^

Proper visualization of the capitellar fragment is sometimes not possible in the routine views of the elbow and a radial head- capitellum view may help in better delineation of the fracture personality.^18^ Properly positioned lateral view is essential for diagnosis, with the fracture easily missed if the projection is slightly oblique as per Fowles and Kassab.^8^ A comparative view of the opposite elbow or CT scan will help in diagnosis. A properly taken lateral view usually shows anterior and superior migration of the capitellar fragment. Characteristic finding in the lateral X-ray is the “double-arc sign” because of the sub-chondral bone of the capitellum and lateral part of trochlea.^13^ The sub-chondral bone of the trochlea creates the double arc and when this sign is present it signifies that a part of the trochlea is also involved.^12^

Radiological diagnosis is difficult in a child because the capitellum is not fully ossified and fused before the age of 9-10 years.^5^ Other authors have suggested an oblique radiograph to detect this injury.^5^ In case of difficulty, in interpreting the radiographs, an arthrogram may be done.^5^ Fractures are often missed in the emergency room setting as the outline of the distal end of the humerus is intact. One case was missed in this series in the emergency room. A CT scan delineates the fracture extent more clearly^12^,^15^ and helps the surgeon plan the approach, since, if the fragment is displaced on the medial side, another medial approach may be needed for reduction.

Treatment of type 2 and 3 capitullam fractures can be either conservative or excision of the fragments.^1^ Ochner reported, in 1996, successful outcome of closed reduction of coronal fractures of the capitellum in nine cases with long term follow-up.^6^ In none of our cases closed reduction was attempted even before open reduction. Closed reduction of the fracture can lead to early arthritis, loss of motion of the elbow or instability of the elbow as it is usually a non anatomical reduction.^14^

Excision of the fragment can lead to instability of the elbow.^19^ Excision to prevent avascular necrosis is suggested by few authors.^15^ Fragment excision due to fear of avascular necrosis or redisplacement can lead to radio-humeral osteoarthritis and instability of the elbow.^20^ Alvarez^2^ advocated excision of the fragment in 10 out of 14 cases

Approaches described include lateral approach (Modified Kocher approach),^11^,^15^ posterior approach with olecranon osteotomy.^11^ Sano^11^ advocates olecranon osteotomy approach for proper visualization of the trochlea, but in the present series by retracting the medial structures with a bone lever the entire medial aspect of the trochlea could be visualized. The authors^11^ found the olecranon osteotomy approach useful if the trochlea also need to be fixed. Screws inserted from posterior to the anterior (PA) direction have more bio-mechanical stability than antero-posterior screws and this prevents damage to the articular cartilage.^3^ Moreover, purchase of screw threads in the sub-chondral bone is more in PA directed screws, and splintering of the sub-chondral bone due to countersinking is less.^3^ Lateral collateral ligament has to be preserved during the procedure.^21^

Various internal fixation methods have been described, including K wires,^1^,^15^ 4 mm cancellous screws,^4^,^15^ Herbert screws^1^,^13^,^15^ and absorbable polyglycide pins.^22^ There are also reports of plate fixation of the fracture.^13^,^16^,^21^ Kirschner wires do not provide enough stability for mobilization before fracture healing and also damage the articular cartilage.^1^ The better functional outcome of operative fixation has been documented.^23^

Headless screws can have problems if the patients develop AVN or chondrolysis, because erosion of the radial head is a possibility due to exposed implants.^1^,^20^ This problem is avoided by the 4 mm partially threaded screws, which could be easily removed through stab incisions. Reports of avascular necrosis of the capitellum are very rare.^24^,^25^

Grantham reported an elbow assessment based on stability, pain and range of movements, which is easy to follow.^19^ Excellent - normal stability, no pain and full range of movements, good - less than 10 degree of instability, mild pain and less than 40 degree restriction of range of movements, fair -10-15 degree of instability, moderate pain or 40-60 degree of loss of range of motion, poor - 15 degree or greater instability, troublesome pain, or 60 degree or more of loss of range of motion.^19^

Articular damage is thought to be the reason for residual extensor lag in spite of anatomical reduction and early mobilization.^1^

## CONCLUSION

Type 4 capitellar fractures are less due to rarity of the injury. The importance of noting double arc sign in lateral view X-rays of the elbow and CT scan evaluation preoperatively is emphasized. The results of fixation with non-cannulated AO screws through extended lateral kocher's approach has given good results.. This report is presented though there is no long term follow-up to document post-traumatic arthritis.
